# Round the Hospitals

**Published:** 1919-05-31

**Authors:** 


### ROUND THE HOSPITALS.

At the investiture held in the Quadrangle at Buckingham Palace on May 22 the King conferred the following decorations on members of the nursing services:—Bar to the Royal Red Cross, Matron Johanna Clay, Q.A.I.M.N.S. (retired); Royal Red Cross First Class, Matron Nellie Blew and Assistant-Matron Mary Graham, Q.A.I.M.N.S.; Sister Alice Milburn, T.F.N.S.; Matron Isobel Alexander, South African M.N.S.; Royal Red Cross Second Class, Sister Edith Beedie, Sister Caroline Crawford, Sister Irene Denson, Sister Catherine Ferguson, Sister Mary Fletcher, Sister Ada Gabriel, Sister Olive Niles, and Sister Helen Ripper, Q.A.I.M.N.S.R.; Assistant-Matron Lilian Noble, Sister Annie Bell, Sister Daisy Hutchings, Sister Elizabeth Jenkin, and Sister Kathleen Topham, T.F.N.S.; Matron Jane Peebles and Sister Nora Rutledge, Civil N.S.; Sister Henrietta Auden, B.R.C.S.; Miss Nathalie Dodd, St. John's A.B.; Sister Lucy Boughton and Sister Lucy McIntosh,

**ROUND THE HOSPITALS—(*continued*).**

Australian A.N.S.; Miss Elsie Buller, the Lady Lilian Digby, Miss Edith Dimond, Miss Madge Kitchener, Miss Elizabeth Macmillan, Sister Rachel Slocock, and Miss Mary Stafford, V.A.D. His Majesty conferred the Military Medal on Miss Nellie Dewhurst, V.A.D., and Miss Stella Dickson, First-Aid N.Y.

THE PRINCE OF WALES, in accepting the position of President of St. Bartholomew's Hospital, has thrown himself heartily into the effort for providing the Nurses' Home. He has sent a message through the Hon. Sir Sidney Greville to Lord Sandhurst, the treasurer, expressing his keen interest in the proposed new quarters for the nurses. "After having seen the noble self-sacrificing devotion displayed by the nurses throughout the war, the Prince feels that the utmost should be done to provide for their comfort when working in hospitals at home." All who are concerned in the undertaking are working with enthusiasm in order to secure the necessary £150,000 this year, and the new President's zeal in the good cause is very stimulating.

DR. AND MRS. WAINWRIGHT, of Henley-on-Thames, acknowledge with profound gratitude through the medium of the Press the plentiful kindness and sympathy shown by the public on the occasion of Miss Edith Cavell's funeral. The letters they have received are too numerous to reply to at once, but they hope to reply as soon as possible. They convey their sincere thanks to the members of the Committee formed by the Anglo-Belgian Union and the Edith Cavell Home of Rest for Nurses for the admirable arrangements made in connection with the ceremony.

VOTING-PAPERS for the new Council of the College of Nursing must be posted next Monday, and it may not be amiss to remind members of the College who desire to see themselves represented by nurses who do not occupy positions of authority that this cannot be brought about unless they take the precaution of voting for the private nurses, district nurses, and sisters whose names will be found in the voting lists. Needless to say, the election of matrons and superintendents is just as democratic as the election of private individuals, for the spirit of democracy lies in spontaneous choice by the body of members. It is, however, very desirable that all grades and branches of the Nursing Service should find a place on the Council.

MEMBERS of the College ought to take particular note of the remarks in the Treasurer's report relating to the cost of registration. The items dealing with this branch of the College work are already very heavy, even before the publication of the Register, the cost of which will probably not fall far short of £1,000. Thus the salaries in connection with registration amounted in 1918 to £621, the stationery and printing to £455, and the postage to £300. In view of the controversies which have arisen in regard to the registration fee these facts are significant. To fix an over-high registration fee will in all probability lead to the exclusion of a considerable proportion of nurses from the benefits of State registration, and this would lessen the value of registration to all. It will, perhaps, have to be recognised that it is more important to the profession generally than even to the individual nurse that all should be registered.

THE joint scheme of training for nurses agreed upon at Liverpool by the Royal Infirmary, the Royal Southern Hospital, the David Lewis Northern Hospital, and the Stanley Hospital marks a new and important step forward in federation. It aims at lessening the disabilities of the special hospitals in this district in regard to completing the training of their probationers. It is a hard matter to expect women, after two years' training in a special institution, to begin all over again and serve four years before obtaining a certificate as a fully-trained nurse. Under the joint scheme the above-named Liverpool hospitals undertake to receive a limited number of junior nurses who have completed two years service in certain selected special hospitals, and give them a certificate of full training after they have passed their final examination and completed three years' service. The scheme is based to some extent on that already at work between the Great Northern Central Hospital and the City of London Hospital for Diseases of the Chest. It should certainly be advantageous to both classes of institution. On the one hand the organisation of the special hospitals will be considerably strengthened by the prospect held out to promising students of obtaining a full nursing certificate when their course is over on easier terms, and on the other hand the general hospitals stand to gain by the admission of junior nurses who have already received a careful training in the performance of some part at least of their duties. There can be little doubt this system will extend to other parts of the country.

A STRONG plea for improved conditions of pay in Queen Alexandra's Royal Naval Nursing Service appears in the *Times* under the initials S.M.O. It is well known that the question of pay and gratuities in this service is under consideration at the Admiralty and that considerable alterations will shortly be announced. The Council of the College of Nursing did a public service in drawing the attention of the Admiralty to the need for reforms. It is high time that this comparatively small, but exceedingly honourable branch of the nursing service should be raised to a level as regards enrolments with the sister military nursing service. As revised in 1911, the scale of pay for the three grades, head sisters, superintending sisters, and nursing sisters, did not compare badly with that of the Army nurses. It is the war conditions of pay which need overhauling. S.M.O. calls attention to the fact that the men who work under the sisters receive something like twice the amount of pay, and even a "sick berth ration" will get far more pay than a sister. This is a condition of things which calls for prompt re-adjustment.

**ROUND THE HOSPITALS—*(continued).***

THE revised scale of working hours and salaries at St. Mary's Hospital, Paddington, bears witness to considerable reconstructive ability on the part of the management. Throughout the establishment the working hours have been reduced to 56 per week, an average eight-hour day, necessitating a considerable increase of the staff. The direct increases in pay take effect principally, as is only just, in raising the value of the sisters' posts. Whereas formerly the ward sisters received £40, rising by £5 increments to £60, they now commence at £50 and rise to £80. The administrative sisters are to receive £75, £85, and £90 respectively, an addition of £20 in two cases, and £15 in the third case to former salaries. Probationers are dealt with in a wise and liberal spirit. In their fourth year all are to receive midwifery training in a lying-in institution to qualify them for the C.M.B. certificate, the monetary value of the course being £21. This gives them a greater advantage than any mere rise in salaries, and is likely to attract a good class of probationer. Reckoning this in, the salaries for the four student years now total £95 in lieu of £87 on the old scale. It is a distinct public benefit that nurses should receive this necessary training as part of their regular course.

---

AT the London Homœopathic Hospital the salary of junior sisters has been raised to £50, and that of senior sisters to £60. Some little time ago probationers' salaries were raised to £17, £18, £20 and £28, with uniform, and their hours on duty were reduced. We learn of improvements also at the Dorset County Hospital, where the salary of the ward sisters is now to commence at £50 and rise to £60, the masseuse sister receiving £60-£70. At this hospital the one day rest in seven has lately been instituted. At Ramsgate General Hospital steps have been taken by the matron, with the consent of the board, to reduce the hours of the night nurses. In lieu of twelve hours at a stretch in the wards, from 8.30 P.M. to 8.30 A.M., they are to have two full hours off during the night outside the wards, besides two separate half-hours for meals. There is a prospect here also of initiating the one day in seven rest.

---

AT the annual meeting of the Norfolk Nursing Federation, held under the presidency of the Countess of Albemarle, it was announced that a minimum commencing salary of £80 a year had been agreed upon for district nurses after a year's training in midwifery and general nursing. The Norfolk County Council has sanctioned a grant of £2,000 towards raising nurses' salaries in the county, and practically all the local associations will this year receive a grant in aid of salaries. There is at present a great dearth of candidates for training, but this will disappear all over the country, we feel confident, as district nurses' posts prove better worth having.

---

AT a meeting of the Bermondsey Board of Guardians on May 23 it was announced that there was a shortage of about thirty nurses at their infirmary at Rotherhithe. One of the Guardians stated the reason why probationers could not be obtained was "lack of comfort." It is astonishing that a small strong committee was not formed on the spot to investigate this charge and bring about a thorough reform should it be needed. The same Guardian asserted they were advertising for nurses at 1½d. an hour, and he hoped they would not get them. This is a ridiculous statement in view of the fact that nurses receive full board and allowances. It can only be misleading to the public to contrast the average pay per hour without emoluments of a pupil-nurse undergoing instruction with that of daily skilled women receiving nothing whatever in kind and often living at an extensive distance from their work. An eight-hour day at 1½d. an hour yields about £18 a year, and to this must be added at least £60 a year for the average cost per head of the Nurses' Home. This is not bad for a beginner.

---

THE Nation's Fund for Nurses marches apace. It is not merely that large sums fall in from time to time; it is that workers are busy in many different parts of the country husbanding the small donations which are offered spontaneously with enthusiasm by all who hear the appeal. Lately the secretary has received the following sums from local centres of the College of Nursing; Aberdeen (per Miss Evelyn Hall), £5 9s.; Leicester (per local Hon. Secretary), £25; Cornwall (per Hon. Secretary, Mrs. Patrick), £8 14s.; Leeds, combined effort with Huddersfield (per Miss Edwards), £1,150 18s. 2d. The King has again shown his sympathy with the movement by graciously giving permission for a Garden Party on June 24 at St. James's Palace in aid of the Fund.

---

THE Ministry of Labour is devoting much praiseworthy energy to the task of resettling the demobilised nursing sisters. Between 2,000 and 3,000 sisters are said to be looking for posts, and the Secretary of the Nurses' Demobilisation and Resettlement Committee at 16 Curzon Street, Mayfair, W. 1, appeals to employers and municipal bodies to communicate particulars of any vacant posts. There is not the least doubt that the public needs the services of every demobilised nurse, either for private nursing, institutional departments, or municipal work. The question about which we are not so sure is whether the public is prepared to pay the proper price for these well-tried and valuable workers. The bad tradition that nurses need only a maintenance wage is hard to break. Having food and raiment, let them be therewith content, says the employer, and a few plain words to philanthropic sweaters would do no harm. It will be to the last degree deplorable if the public is induced by hearing how many nursing sisters are on the waiting-list to attempt to keep back the rising tide of remuneration. A rich return awaits the municipal and institution authorities who create in good time posts worthy to attract to their service the waiting sisters.

**ROUND THE HOSPITALS—(*continued*).**

Miss Alice Fitzgerald, R.R.C., of the American Red Cross, has been appointed Chief Nurse for Europe, with headquarters in Paris. She received her war training under the British Red Cross Society, and came to England in March 1917 as the "Edith Cavell Memorial Nurse," to serve with the British Forces during the war. She was transferred to the American nursing service on the entry of the United States into the war, and has done good service in France and Italy. In addition to the Royal Red Cross, she has been awarded the French Medal of Honour, and for services rendered in the Messina disaster received the Italian Red Cross.

Among the celebrations with which the American Red Cross Society will mark Commemoration Day, May 30, it is interesting to learn that a visit to the grave of Florence Nightingale, near Romsey, is included. We are sure also that the grave of Edith Cavell will be honoured by her sisters from the other side of the Atlantic. Six American nursing sisters lie buried over here. Their names and burial places are:—Clara M. Orgren, Colorado Springs, buried at Brookwood; Theresa M. Murphy, Concord N.H., buried at Haslemere; Hattie M. Raithel, Denver Col., buried at Brookwood; Florence L. Athay, Newark N.J., buried at Liverpool; Geneva Cass Stevens, Beecher City, Ill., buried at Liverpool; Tulla Lake Harkey, D.Sc., New York, buried at Winchester. There will be a special memorial service at 11.30 at Brookwood cemetery, where there are 130 American graves, including those of two American nurses.

The Victory Bazaar which was held at the Chelsea Town Hall on May 21 and 22 proved very attractive and successful. It was promoted by the Chelsea, Pimlico, and Belgravia Nursing Association, under the presidency of the Rev. R. Hudson, Mayor of Chelsea. The Association, in spite of all the good work accomplished during the past year, has not been very generously supported in this rich neighbourhood, and has issued an appeal for help to carry on its ministrations among the sick poor.

The ragging case at the Bermondsey Infirmary has ended as was expected. Each of the five assailants has been fined 40s. and costs on the charge of assault. It came out in evidence that the four probationers began their training thirteen months ago, some few weeks before Miss Russell arrived, and that they were aggrieved at being twice reported by her. Mr. Percy Robinson, their solicitor, stated that the Guardians had made full inquiries and repudiated the suggestion that patients had been neglected. Mr. Gill, in imposing fines, said the complainant had been subjected to very great indignity, and that there was no reason or excuse forthcoming. He could not deal with the case under the Probation Act because it was not a trivial case, and there were no extenuating circumstances. We forbear comment on a case which has created unmixed displeasure in the nursing world.

Congratulations to Miss Evelyn May Kealy, Westminster Hospital, who has been the recipient of the "Matron's Prize" for her practical ward work. This is kindly given each year by the vice-Chairman of the hospital, Mr. Vaux Graham.

The Jewish Maternity Home, Nursing and Sick-Room Help Society held its annual meeting on May 21 at the house of Sir Marcus and Lady Samuel and produced an excellent record of work accomplished. The increased need for lying-in homes is shown by the fact that the maternity home with its 11 beds and record of 275 patients admitted to the wards was compelled to reject more applicants than it received. The Society, like most Jewish philanthropic undertakings, is run on very good lines, and has attracted attention from Government departments and municipal bodies. Its organisation and teaching of sick-room helps is particularly good and has enlisted a grant from the Local Government Board. A dental clinic has been added to the infant welfare centre, and an observation nursery has proved of great value in demonstrating to mothers the need for care, cleanliness, fresh air, and common sense in the rearing of their offspring.

That the number of tuberculosis patients is higher than was estimated when the National Insurance Act was passed, and far exceeds the accommodation available for treatment, has long been known. The Sanatorium Benefit Sub-Committee is badly hampered for lack of money, hundreds if not thousands of discharged soldiers are left without any chance of institutional treatment; and during the transfer of powers to the Ministry of Health little action can be taken to remedy matters. In these circumstances great relief might be afforded to patients were they able to obtain without delay or difficulty the loan of an open-air shelter where they could sleep, have their meals, and pursue such avocations as do not call them into the open. Under the treatment of local practitioners and with the help and advice of district or village nurse recovery lies within reach of all in the primary stages who will submit themselves to the regimen, and this without the cost and delay involved in procuring sanatorium treatment. It cannot too often be insisted on that though country surroundings make the consumptives' prospects in villages less despairing than in towns, yet the senseless diamond pane casement, opening but a few inches over a third part of its narrow space, turns the ordinary rustic cottage into a death-trap for all attacked by tuberculosis.

The Board of Management of the County Hospital, Lincoln, have made arrangements by which a fourth-year's training will be given to approved nurses, consisting of massage for six months, and housekeeping, laundry work and private nursing for six months. It has also been arranged that members of the nursing staff are to have three hours off duty daily, and one day each week free. There will be a few vacancies for pupils, resident and non-resident, in the massage school.

